# Toward structured fellowships in fetal neuroimaging

**DOI:** 10.1111/aogs.70078

**Published:** 2025-10-12

**Authors:** Shiri Shinar, Elka Miller

**Affiliations:** ^1^ Ontario Fetal Center, Department of Obstetrics and Gynaecology, Division of Maternal Fetal Medicine Mount Sinai Hospital, University of Toronto Toronto Ontario Canada; ^2^ Ontario Fetal Center, Department of Diagnostic and Interventional Radiology, the Hospital for Sick Children University of Toronto Toronto Ontario Canada

Fetal neuroimaging has become one of the most dynamic and demanding areas within maternal–fetal medicine. Advances in ultrasound resolution, the widespread availability of fetal MRI, and the integration of genomic testing have transformed the way anomalies of the developing brain are detected, interpreted, and communicated to families. These advances raise a fundamental question: how can clinicians gain comprehensive expertise in the diagnosis, prognostication, and management of fetal neurological conditions?

## 1 CHALLENGES IN FETAL NEUROIMAGING

The first challenge lies in imaging itself. At the foundation of fetal neuroimaging is neurosonography, defined by ISUOG as a dedicated, multiplanar diagnostic examination of the fetal brain and spine in pregnancies at high risk for neurological malformations.^1^ This requires technical mastery in both transabdominal and transvaginal approaches, as well as growing competence in three‐dimensional imaging. Yet even with the most skilled hands, ultrasound has limitations, and fetal MRI is increasingly used to complement sonography. Interpreting prenatal MRI, however, is a specialized skill in its own right. Few maternal–fetal medicine specialists or radiologists receive structured and formal training in acquisition protocols or in the nuanced interpretation of congenital and acquired brain abnormalities. The result is highly variable expertise, dependent largely on local mentorship or exposure.

Interpretation of findings constitutes the next major hurdle. Imaging phenotypes are often complex, subtle, and overlapping across different etiologies. A finding that suggests a vascular pathology may mimic infection or genetic disease, while subtle and focal cortical malformations may escape recognition altogether without careful review. Furthermore, even after a diagnosis is reached, terminology in the literature remains inconsistent, with definitions used inaccurately and interchangeably (i.e. anomalies in the spectrum of Dandy–Walker, or those involving the corpus callosum^2^). This lack of standardization hampers meaningful data comparison and undermines the reliability of prognostic assessments.

These challenges are compounded by the timing of assessment. Gestational age influences the spectrum of findings that can be visualized, as many anomalies, particularly those associated with cortical development, evolve over time and require follow‐up to clarify their progression and clinical significance. Early fetal MRI can provide valuable complementary information to ultrasound, but is often limited by motion artifacts that obscure subtle abnormalities. Later sonographic and MRI assessments may yield more definitive insights; however, these options may not be available in settings where termination of pregnancy is legally restricted beyond certain gestational age thresholds.

Postmortem imaging is an emerging technique that adds further dimensions^3^ clinicians must learn to incorporate. This means that expertise in fetal neuroimaging today requires fluency not only in ultrasound and MRI, but also an understanding of pathology and developmental neuroscience.

A further complicating factor is the interobserver variability in CNS imaging. Even among experienced practitioners, agreement on subtle posterior fossa or cortical anomalies is inconsistent, and retrospective review often reveals lesions that were present prenatally but not recognized and vice versa.^4^, ^5^, ^6^ Concordance between prenatal and postnatal findings depends on expertise and varies considerably in the literature.^4^, ^7^, ^8^, ^9^, ^10^ Reported rates vary, but some studies demonstrate a relatively high frequency of discordant findings on pre and postnatal imaging, particularly for posterior fossa anomalies where discordance may be as high as 40%^11^ and for cortical anomalies, with discrepancies frequently reported and only moderate agreement between prenatal and postnatal MRI assessments.^7^ The burden of missed or mischaracterized diagnoses highlights the ongoing challenges in accurately detecting and characterizing fetal CNS anomalies, with each missed diagnosis representing not only a lost opportunity for accurate prognostication and counseling, but also a potential compromise in informed parental decision‐making.

Prognostication represents perhaps the greatest difficulty in fetal neuroimaging. For families, the critical question is not simply what anomaly exists, but what this means for their child after birth.^8^, ^9^, ^12^ The intrinsic plasticity of the developing fetal brain further complicates prognostication in a wide range of malformations and disruptive lesions. In addition, as fetal neuroimaging is still emerging and a proportion of cases result in termination of pregnancy; robust long‐term outcome data for many anomalies are scarce, further challenging prognostication.^13^, ^14^ Counseling usually comes from neonatologists, pediatricians, pediatric neurologists, neurosurgeons, and developmental specialists. Yet in the interval before formal consultation, parents turn first to their obstetrician, who must be prepared to provide preliminary prognostic guidance. Without this, families may be left in uncertainty, often resorting to unverified online sources that amplify fear and confusion. The difficulty is that obstetricians and radiologists rarely have detailed knowledge of longitudinal effects on children. Unlike pediatric neurologists, they cannot easily correlate prenatal imaging findings with long‐term neurodevelopmental outcomes. As a result, counseling families about prognosis can feel speculative, even when imaging itself has been expertly performed.

The scientific landscape continues to evolve, adding further complexity. Genomic medicine has introduced powerful tools for diagnosis. Whole‐exome and whole‐genome sequencing are now integrated into prenatal care, with diagnostic yields approaching 44% in multisystem anomalies and nearly 60% in complex brain anomalies.^15^, ^16^ Yet interpreting these results is not straightforward. Variants of uncertain significance, susceptibility alleles, and incomplete penetrance frequently complicate interpretation. The result is that families may receive an “abnormal” genetic result without a clear link to prognosis. Without integrated expertise across imaging, genetics, and neurodevelopment, such findings risk creating more confusion than clarity.

These multiple challenges underscore the need for a multidisciplinary team—the fetal neurology clinic (FNC). An effective FNC brings together maternal–fetal medicine specialists, pediatric neurologists, neuroradiologists, neurosurgeons, neonatologists, geneticists, perinatal pathologists, and allied health professionals, including psychosocial support.^17^ Without such collaboration, families often experience fragmented care, with one clinician responsible for imaging, another for genetic counseling, and yet another for developmental prognostication. Although specialists may at times meet for in‐person case discussions, these nonconcurrent multidisciplinary meetings cannot replace the value of a dedicated FNC, where families meet all relevant specialists together, receive a unified message, and benefit from real‐time dialogue between disciplines.^18^ Studies have shown that expecting mothers would value the *coordinated* involvement of different specialists.^19^, ^20^ Indeed, the multidisciplinary model allows families to receive coherent and integrated counseling. Expert opinion from pioneering FNCs suggests that these teams not only improve care but also provide an ideal platform for structured education.^7^, ^17^, ^21^, ^22^

Despite these formidable requirements, no formal training programs or accredited fellowships in fetal neuroimaging exist. This stands in sharp contrast to other areas of medicine. Pediatric cardiologists complete structured fellowships in fetal cardiology, geneticists pursue accredited programs in reproductive and prenatal genetics, and neuroradiologists undergo years of focused subspecialty training. In fetal neuroimaging, by comparison, expertise is acquired through piecemeal exposure, international mentorship, or self‐directed learning—opportunities available only in select centers. This lack of standardization perpetuates uneven expertise across regions and institutions.

Taken together, these challenges demonstrate the remarkable breadth of expertise expected from clinicians in fetal neuroimaging. Yet the infrastructure to train such experts does not exist. While no standardized fellowship currently exists, important progress has been made through published sonographic guidelines for brain assessment^1^, ^23^ and promising training models (OPUS, FaBiAN^24^). High‐functioning FNCs provide platforms for learning; international CME programs, webinars, and registries offer partial standardization and valuable data. Yet these remain fragmented and lack formal recognition.

## 2 NEXT STEPS TOWARD FORMAL TRAINING IN FETAL NEUROIMAGING

The best way forward is to build structured training in neuroimaging on the foundation of multidisciplinary FNC. It has previously been suggested that fetal neurosonography should be an integral part of the MFM fellowship.^17^ While exposure and preliminary performance of neurosonograms should be part of the MFM fellowship, it is not feasible to gain this skill along with fetal MRI in such a short time period. It is time for formalizing fellowships in fetal neuroimaging. Such programs should span 12–18 months and should be designed for MFM specialists, who are already skilled and experienced sonographers, or for radiologists, who have already achieved core competency in obstetric ultrasound or neuroimaging. High‐functioning FNCs provide the optimal platform for the training of future specialists, bringing together imaging, diagnosis, prognostication, and counseling.^17^, ^22^

Multidisciplinary commitment is essential. Fellowships must be rooted in institutions where pediatric neurology, neuroradiology, genetics, neonatology, pathology, and psychosocial services are fully engaged. Without this collective commitment, any fellowship risks becoming siloed and incomplete. Training would begin with fetal neurosonography, with fellows gaining hands‐on experience in transabdominal and transvaginal brain scans, mastering multiplanar 3D/4D techniques, and reviewing a high volume of cases within a FNC. Fetal MRI forms the second cornerstone, with trainees supervising examinations, learning appropriate sequence selection, and interpreting brain and spine abnormalities in depth.

Rotations in related disciplines provide the crucial link between imaging and outcomes. Pediatric neurology, neurosurgery, and developmental pediatrics teach prognostication; genetics introduces CMA, exome, and genome sequencing in counseling; and neuropathology adds another essential dimension, with fellows exposed to macroscopic brain improving understanding of embryology, as well as to newer modalities such as postmortem imaging. The latter can help improve in vivo image interpretation and deepen understanding of brain malformations. These rotations together will help the functionality of the FNC, providing the trainees with a solid understanding of the roles and perspectives of their colleagues. Importantly, the training is not intended to produce a fetal neurologist proficient in image acquisition and interpretation, but rather to equip the fetal medicine specialist/radiologist with a deeper understanding of pathology and neurology, thereby enhancing diagnostic accuracy and prognostication.

Equally important is communication: fellows must learn to deliver difficult news and provide anticipatory guidance. Participation in joint consultations within the FNC ensures these skills are developed in a real‐world, multidisciplinary setting. Research and academic development need to be integrated. Fellows should contribute to registries, publish research, and engage in international teaching and collaborations—ensuring they not only acquire expertise but also help advance the field globally.

Endorsement by authorities and international societies will be necessary for these fellowships to succeed. Accreditation by professional societies would provide credibility, ensure consistency across institutions, and help establish fetal neuroimaging as a recognized subspecialty. Such endorsements would also encourage funding bodies and training authorities to support program development. Finally, performance must be evaluated. Competency‐based benchmarks should be defined, and outcomes should be tracked, not only in terms of imaging accuracy but also in the quality of counseling provided to families.

## 3 CONCLUSION

Fetal neuroimaging is an evolving subspecialty of MFM and neuroradiology, yet the expertise it requires goes far beyond traditional training. Imaging, interpretation, prognostication, and multidisciplinary collaboration are cornerstones; however, all present formidable challenges. These cannot be met through piecemeal education. Structured fellowships, rooted in FNC and endorsed/regulated by international societies, are urgently needed. Only through such fellowships can we ensure that families facing a fetal brain diagnosis receive the expert, comprehensive, and compassionate care they deserve.

## AUTHOR CONTRIBUTIONS

Shiri Shinar (Obstetrician/Maternal‐Fetal Medicine) and Elka Miller (Pediatric Neuroradiologist) jointly conceived, drafted, and revised the editorial. Both authors approved the final manuscript.

## FUNDING INFORMATION

The authors did not receive any funding for this research.

## CONFLICT OF INTEREST STATEMENT

The authors have no conflict of interest.

## Data Availability

Data sharing not applicable to this article as no datasets were generated or analysed during the current study.
